# Primary treated versus referred trunk and extremities soft tissue sarcomas: comparative analysis of initial treatment impact on disease control

**DOI:** 10.3332/ecancer.2025.1933

**Published:** 2025-06-26

**Authors:** Walter S Nardi, Lucía Aragone, Sergio D Quildrian

**Affiliations:** 1 Sarcoma and Melanoma Unit, Department of General Surgery, British Hospital of Buenos Aires, Perdriel 74, CABA, Buenos Aires 1280AEB, Argentina; 2 Department of General Surgery, British Hospital of Buenos Aires, Perdriel 74, CABA, Buenos Aires 1280AEB, Argentina

**Keywords:** soft tissue sarcoma, guidelines, local relapse, centralisation, extremities

## Abstract

**Introduction:**

Soft tissue sarcomas (STS) are rare and aggressive tumours that require a complex multimodal treatment at referral centers. However, they are often misdiagnosed and subsequently improperly treated at non-specialised centers. A multidisciplinary approach is mandatory for these tumours, involving multiple specialties. Therefore, management should be carried out in reference centers for STS. We aimed to compare oncological outcomes of trunk and extremities STS primarily treated at a reference center versus those referred after initial surgical treatment elsewhere.

**Methods:**

All patients with diagnosis of trunk and extremities STS between January 2010 and May 2024, primarily operated at our center or referred after treatment elsewhere, were included. Visceral, retroperitoneal/pelvic, spermatic cord and head/neck STS were excluded, as well as desmoid tumours and dermatofibrosarcoma protuberans subtype. Demographic data and tumour characteristics were evaluated (location, size, French Federation of Cancer Centers Sarcoma Group grade, neo/adjuvant treatment) as well as primary surgery outcomes (R classification). The cohort was divided into two groups: G1 (primary-resection group) and G2 (referred group). Overall survival (OS), local recurrence-local relapse-free survival (LRFS) and distant metastasis-free survival (DMFS) were compared between groups.

**Results:**

A total of 102 trunk and extremities STS underwent surgical resection on the mentioned period, out of which 49 were primarily resected (G1) and 53 had previous resections elsewhere (G2: 33 referred for recurrent tumours and 20 referred after inadequate excision). Data on grade was available for 91 lesions and 67% (61/91) were high-grade, with no significant differences between groups. The two groups had statistically significant differences in median tumour size (G1: 9.5 cm versus G2: 4 cm; *p* < 0.001), preoperative radiotherapy (6 versus 0; *p* = 0.01) and complete resection margins at first surgery (G1: 46 versus G2 3; *p* = 0.0001). All patients in G1 had macroscopic complete bloc resections (94% R0 and 6% planned R1 margins). In G2, residual disease was present in 35% (7/20) of the re-resection specimens. All recurrent tumours had macroscopic complete resections at our center (80% R0 and 20% R1 margins). Discussion within a specialised multidisciplinary tumour board was also significantly different between both groups of patients (98% versus 3.8%; *p* < 0.00001). Three-year LRFS was found to be significantly better when primary surgery was performed at a reference center, with 91% versus 32% (log-rank *p* < 0.0001). No differences were seen in 3-year DMFS (68.7% versus 72.6%, p = 0.55) and OS (85.3% versus 88.1%, *p* = 0.72). Positive resection margins at first surgery correlated with worse LRFS (OR 23.1, *p* = 0.01).

**Conclusion:**

Better local control was achieved in patients initially treated at our center. Being surgical margin status is the primary prognostic factor for LRFS, STS should be treated in referral centers where a multidisciplinary approach and proper oncologic resections following sarcomas guidelines recommendations are standard of care. Hence, the importance of a prompt referral even before any intervention in the event of a suspected diagnosis.

## Introduction

Soft tissue sarcomas (STS) are a rare heterogeneous group of malignant tumours of mesenchymal origin with an estimated incidence of four new cases/100.000/year and encompassing more than 100 histological subtypes with different patterns of clinical behaviours [1, 2]. Trunk and extremity sites are the most frequent locations in STS and represent approximately 55% of all STS. Current clinical practice guidelines (CPGs) recommend specialised multidisciplinary teams to carry out the treatment of sarcoma patients after multidisciplinary tumour board meeting (MDTB) discussions in reference centers. Different studies have already shown the benefits of this management [3–6]. Importantly, the quality of primary surgery (e.g., *en* bloc wide excision with histologically negative margins) is a major prognostic factor for disease control in localised STS with curative intent [1]. However, because of their rarity, they are usually misdiagnosed and hence not treated accordingly. Sometimes, even patients who underwent primary surgery elsewhere without previous biopsy or pre-operatory imaging (known as ‘whoops procedure’) [7, 8] are not referred until a tumour recurrence.

CPGs on STS management strongly recommend early referral before any treatment in the case of a suspected diagnosis of sarcoma [1, 9]. Different clinical scenarios should raise awareness to make a prompt counsel to specialised tertiary centers [10]. In some countries, such as France, UK or Scandivadian ones, sarcoma care is centralised and management must be carried out in designated high-volume reference centers [3, 11]. Nevertheless, many sarcoma patients are still primarily treated in non-specialised hospitals or clinics without following the recommendations of CPGs.

This study analyses the impact of the first surgery performed outside a reference center compared to the surgical treatment given in a specialised sarcoma center.

## Methods

All adult (age ≥ 18 years) patients with confirmed localised STS of trunk and extremities initially managed at our center or referred after surgical treatment elsewhere between January 2010 and May 2024 were included.

In the analysis, referred patients were included only if a complete history (including surgical and pathologic reports) could be obtained. Head and neck sarcomas, visceral sarcomas, retroperitoneal/pelvic sarcomas, spermatic cord sarcomas, desmoid tumours and dermatofibrosarcoma protuberans were excluded. Data on the initial tumour size was retrieved from the pathologic report and was tabulated in cms. The R classification system was used to define the quality of surgical margins throughout all patients of the study [12]. Macroscopically complete resection with no tumour cells in resection margins was considered R0. If microscopic tumour cells were identified at the resection margin or at <1 mm from the inked surface was considered R1. Finally, R2 was considered as a macroscopic residual disease or tumour rupture in the first surgery. The histo-pathological grading was calculated using the French Federation of Cancer Centers Sarcoma Group grading [13]. All diagnosis of sarcoma performed outside were reviewed by our expert soft tissue pathologists.

As a specialised unit in the management of STS, we treat our patients based on the recommendations of CPGs. In the present work, we specifically report some points of interest within sarcoma guidelines (pre-operative biopsy, proper pre-operative staging images, discussion at a specialised MDTB, complete pathologic report and treatment at a referral center) and then analysed its impact on the disease’s natural history. Pre-operatory correct staging was defined if contrast-enhanced chest, abdomen and pelvis computed tomography and magnetic resonance imaging of the affected site were performed.

For the purpose of the analysis, the cohort was divided into two groups depending if primary surgery was performed at our center (Group 1) or operated elsewhere in non-specialised centers and then referred (Group 2). For survival analysis, the date of first surgery was considered the starting point. Local relapse-free survival (LRFS) was computed to the date of last follow-up or date of first local recurrence (LR). Distant metastasis-free survival (DMFS) was computed to the date of last follow-up or date of systemic progression. Overall survival (OS) was considered to the date of last follow-up or death. Also, the length of follow-up was calculated from the date of first surgery to the last follow-up or death. All patients were discussed in our sarcoma MDTB. Re-excision surgery after inadequate margins outside a reference center is systematically considered and offered to patients (mainly if: high-grade [G2-3], deep and/or >5 cm).

The primary end-point of our study evaluated the impact of primary surgery on local control in terms of LRFS and described the risk factors associated. Second, we analysed DMFS and OS.

### Statistical analysis

Statistical analysis was performed using Jamovi Computer Software (Version 2.4.12.0), Sydney, Australia. Continuous quantitative data are described using mean and standard deviation or median and interquartile range (IQR). Qualitative data are described as absolute numbers with percentages. Pearson’s chi-square test (or Fischer’s exact test) was used for univariate analysis. While binomial logistic regression and the cox proportional hazards model were used for the multivariate analysis, including only parameters significant (*p* < 0.05) in univariate analysis. We included in the analysis classical prognostic factors described for sarcomas (age, size, grade, histotype, site, management and surgery at referral center). The Kaplan–Meier method was used for survival analysis and compared using the log-rank test.

## Results

During the study period, 49 patients with primary localised trunk and extremities STS were primarily resected at our center (Group 1). In the same period, 53 referred patients were also operated after a first previous resection elsewhere (Group 2); 33 had recurrent tumours and 20 were immediately referred after initial inadequate excision. Hence, 102 patients constitute the total study cohort. Demographic and tumour characteristics by groups are presented in Table 1.

Overall, patients operated primarily at our center had larger tumours (*p* < 0.001) with a median size of 10 versus 4 cm of referred patients. Data on grade was available for 91 lesions and 67% (61/91) were high-grade, with no statistically significant differences between groups. Remaining demographic or histopathology data of the groups are described in Table 1.

Pre-operative biopsy (core or incisional) was performed in 47 of 49 (96%) patients at our center versus 2 of 53 (3.8%) patients operated outside (*p* < 0.00001). Presentation and discussion in specialised MDTB were also significantly different between both groups of patients (98% versus 3.8%; *p* < 0.00001). Adequate pre-operative staging images had been performed in 46 of 49 (94%) patients of group 1 versus 2 of 53 (3.8%) patients of group 2 (*p* = 0.0001). At our center, more patients were managed according to CPGs before surgery, as expected. See oncologic outcomes by groups in Table 2.

When comparing the quality of surgery, we found that complete resection margins (R0 rate) at first surgery were far superior to those of patients operated in non-specialised centers (94% versus 5.6%; *p* = 0.0001). All patients in G1 had macroscopic complete en bloc resections (94% R0 and 6% planned R1 margins). Conversely, the rate of R2 resection was higher in non-specialised centers (34% versus 0% at our center).

Overall, in 35.8% (19/53) of patients operated outside resection margins were not possible to analyse. Interestingly, in 19% (10/53) of pathologic reports did not inform the R status; and in 17% (9/53) data were unknown. Of the patients with initial unplanned excisions (‘*whoops surgery*’) (*n* = 20), 55% (11/20) had incomplete resection margins and in 25% (5/20), margins were not analysed. Residual disease was present in 35% (7/20) of the re-resection specimens. After re-resection surgery, the final R-status was R0 for all patients (20/20). Also, all recurrent tumours (*n* = 33) had macroscopic complete limb-sparing resections at our center (80% R0 and 20% R1 margins).

**Figure 1.**
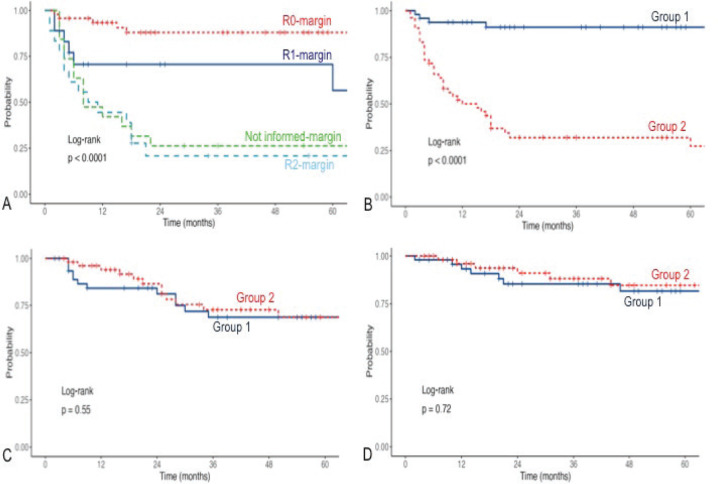
(a): LR free survival according to margin status after first surgery. (b): LR free survival according to groups. (c): DMFS. (d): OS.

We then analysed prognostic factors for relapse after the initial surgery, either in our center or outside. In total, 44 patients had an LR: 33 presented with the recurrent tumour after initial treatment outside, as stated before, 6 had an LR after R0 re-resection surgery and 5 after initial surgery at our center. Median time to first LR was 5 (IQR, 2.5–46) months versus 5 (IQR, 3–18) months for patients referred for a recurrent tumour. Basically, lack of adherence to sarcoma guidelines and prior inadequate management elsewhere were associated with LR as well as incomplete resection margins at first surgery (all *p* < 0.0001) on univariate analysis (Table 3). Margin status R0 was found as a significant risk factor for LR in multivariate analysis (OR: 23.1, 95%CI 6.6–80.6, *p* < 0.001) (Table 4). No significant differences were observed in univariate analysis when considering DM (Table 5). See LR survival estimates according to margin R status on Figure 1a. Not informed margins and R2-margin status had similar LRFS estimates.

We found statistically significant differences in 3-year LRFS when comparing G1 versus G2 (91.1% (95% CI, 83%–99.9%) versus 32% (95% CI, 21%–48.5%), log-rank *p* < 0.0001) with a median follow-up of 40 (14.5–70) and 31 (12–57) months, respectively (Figure 1b). No differences in 3-year DMFS (68.7% versus 72.6%, *p* = 0.55) and OS (85.3% versus 88.1%, *p* = 0.72) (Figure 1c and d).

## Discussion

In the present study, we investigated the impact of primary surgery in a reference center compared with non-specialised centers, with a 3-year LRFS significantly higher of 91% (versus 32%). The other oncologic outcomes analysed were similar.

Management of most oncological entities is guided by CPGs. They are evidence-based recommendations prepared by leading experts to help patients with the best care options available. Specifically in the sarcoma field, multidisciplinary treatment in high-volume reference centers is strongly advised and also improves adhesion to CPGs [14–16]. In the multicentric French study (which included sarcomas and intermediate malignant soft tissue tumours of all locations), over 50% of the 29,497 patients had no previous pre-operatory biopsy, no adequate imaging or were operated outside reference centers [3]. In our study, most of the patients treated elsewhere (and referred after a first LR) were not managed following sarcoma guidelines, denoting a clear problematic given that this subgroup represents more than half of the cohort. Additionally, discussion within an MDTB was also very low compared to our center. Debate in MDTB is the best way to deliver complex care to cancer patients. In rare cancers such as sarcoma, even more, being associated with better fulfillment of CPGs, better quality of surgery and relapse-free survival [4].

Quality of initial surgery (i.e., status of surgical margins) is of utmost importance for local disease control being the main prognostic factor [17–20]. When the first surgery was performed at our center, 94% of the patients had R0 margins and 6% (3/49) had planned R1 margins close to critical structures such as vessels, nerves or bone. Of these latter patients, one was a WDLPS with confirmed pre-operative MDM2 amplification and in the other two, neoadjuvant radiotherapy was performed to offset the negative prognostic impact of the anticipated R1 margin [21–23]. Conversely, the rate of R0 resections outside was much lower, with a significant 34% of R2 resections. Besides, almost 36% of the margin status in the referral subgroup were not possible to analyse, mainly due to a lack of reports. In these patients without documented margins on the pathologic report, we could also see a higher risk of local relapse similar to R2-resections. Patients operated on in our center had worse prognostic characteristics with larger and deeper tumours. Grade was equally distributed. Despite this, the quality of surgery was better and this was translated into better local control. Different studies have demonstrated a lower rate of positive tumour margins and better LRFS when primary surgery is performed at an expert center with MDTB pre-operatory discussions [4, 24, 25].

As said previously, the referred patients in our study represent 52% of the cohort and 62% of them were referred only after a first recurrence. This is another important topic for improvement since non-experienced physicians subsequently managed those patients after the diagnosis instead of referring them to experienced centers. It can be hypothesised that they could have benefited with other multimodal strategies. Inexperience in primary care leads to delayed referrals or inadequate excisions (38% of the referred subgroup). Early referral to an expert center should be done to ensure an appropriate multimodal treatment. In fact, as soon as sarcoma is suspected clinically, it is better that further diagnostics be made by a specialised sarcoma surgeon [10]. Implementing sarcoma guidelines has been associated with more early referrals to expert centers, even before excision biopsies [1].

Organisation and centralisation of sarcoma care is not a new concept. Development of specialised centers/units with multidiscipliary management has evolved with the support of strong health care policies and scientific societies. For example, the German Cancer Society demands multiple specialities on a regular MDTB for the certification of sarcoma centers in Germany [26]. In France, the creation of NetSarc database (French clinical reference network for soft tissue and visceral sarcomas) and approval in 2014 by the French National Cancer Institute have proven successful and transformational, as patients treated within the network have seen better outcomes. Nowadays, NetSarc is composed of 28 reference centers. The UK guidelines for the management of STS stipulate precise indications of how sarcoma services should be formed and of the referral pathways in case of suspected sarcoma [27]. However, most parts of the world have not been able to adopt these strategies so far, even when there is growing evidence showing a clear benefit. Latin America faces several challenges that would help optimise care and resources. Clinical and referral pathways are not well standarised as well as recognition of reference centers [28].

This study has several limitations: it is a retrospective single-center study with a limited follow-up and number of patients. It is planned to expand the numbers with the inclusion of other sites to improve the analysis of sarcoma care situation in our country. Consequently, a longer follow-up will make the outcome analysis more interesting. We acknowledge how important collaborative efforts and data sharing are in this rare patology. Despite all this, to the best of our knowledge, this is the first national series to analyse and provide updated information on this problematic. We think this retrospective information brings valuable insight and can be the starting point to better healthcare practices in sarcoma.

## Conclusion

When statements proposed in CPGs are fulfilled in reference centers, patients have a greater chance of disease control. Becomes clear enough, even in small series such as ours, that diagnosis, treatment and follow-up care of sarcomas patients should be realised in a specialised unit composed of multidisciplinary experts (sarcoma pathologist included) which meets regularly in a place with appropriate imaging. Prompt referral to a specialised center is imperative if those core principles cannot be accomplished to obtain the best results possible, as demonstrated in different series.

## List of abbreviations

CPGs, Clinical practice guidelines; DMFS, Distant metastasis free-survival; FNCLCC, French Federation of Cancer Centers Sarcoma Group; LR, Local recurrence; LRFS, Local relapse free-survival; MDTB, Multidisciplinary tumour board meeting; OS, Overall survival; STS, Soft tissue sarcoma.

## Conflicts of interest

None.

## Funding

There was no funding for the present study.

## Author contributions

Study concept and design: WSN. Acquisition of data: WSN, LA. Drafting of the manuscript: WSN. Critical revision of the manuscript for important intellectual content: SDQ. Final revision and final approval for publication: WSN, SDQ. All authors read and approved the final manuscript.

## Figures and Tables

**Table 1.** Demographic variables, tumour’s characteristics, treatment modalities and morbidities.

	Primary resection *n* = 49	Referred *n* = 53	Univariate analysis *p*
Sex, *n* (%)			
°Male	28 (57.1)	30 (56.6)	1
°Female	21 (42.9)	23 (43.3)	
Age, median (IQR)	54 (45–71)	51 (39–68)	0.4
Tumor size in cm, median (IQR)	9.5 (5–13)	4 (2.5–6.8)	**< 0.001**
Tumor location, *n* (%)			
°Trunk	13 (26.5)	22 (41.5)	0.1
°Extremities Depth °Superficial °Deep	36 (73.5) 14 35	31 (58.4) 31 22	**0.0014**
Histopathology, *n* (%)			
°WDLPS	5 (10.2)	3 (5.6)	0.4
°DDLPS	9 (18.3)	4 (7.5)	0.1
°MLPS	8 (16.3)	3 (5.6)	0.1
°LMS	9 (18,3)	11 (20.7)	0.8
°UPS	3 (6.1)	12 (22.6)	**0.02**
°MFS	4 (8.1)	7 (13.2)	0.5
°MPNST	3 (6.1)	0	0.1
°SFT	2 (4.1)	1 (1.8)	0.6
°Others*	6 (12.2)	12 (22.6)	0.2
FNCLCC grade, *n* (%)			
°1	16 (32.7)	14 (26.4)	0.5
°2	10 (20.4)	15 (28.3)	0.3
°3	19 (38.8)	17 (32.1)	0.5
°Not informed	4 (8.1)	7 (13.2)	0.5
Reconstruction, *n* (%)			
°Primary closure	35 (71.4)	38 (71.6)	1
°Skin graft	2 (4.1)	1 (1.8)	0.6
°Local flap	3 (6.1)	3 (5.6)	1
°Pedicled flap	1 (2.1)	2 (3.7)	1
°Free-flap	4 (8.1)	4 (7.5)	1
°Mesh	2 (4.1)	5 (9.4)	0.4
°Vascular	2 (4.1)	0	0.2
Perioperative RT, *n* (%)			
°Preoperative RT	6 (12.2)	0	**0.01**
°Postoperative RT	13 (26.5)	1 (1.8)	**0.0003**
Complications (Clavien-Dindo), n (%) Total	7 (14.2)	4 (7.5)	0.3
°IIIa	0	1 (1.8)	1
°IIIb	7 (14.2)	2 (3.7)	0.08
°IV	0	1 (1.8)	1
Months of follow-up, median (IQR)	40 (14–75)	27 (12–55)	0.1

WDLPS = Well differentiated liposarcoma; DDLPS = Dedifferentiated liposarcoma; MLPS = Myxoid liposarcoma; LMS = Leiomyosarcoma; UPS = Undifferentiated pleomorphic sarcoma; MFS = Myxofibrosarcoma; MPNST = Malignant Peripheral Nerve Sheath Tumour; FST = Fibrous solitary tumour; FNCLCC = French Federation of Cancer Centers Sarcoma Group; RT = Radiotherapy

* Others = Fibromixoid sarcoma (3), synovial sarcoma (3), angiosarcoma (2), hemangiosarcoma (2), rhabdomyosarcoma (1), epithelioid sarcoma (1), myofibroblastic sarcoma (1), mixoinflamatory fibroblastic sarcoma (1), dermatofibrosarcoma (1), clear cell sarcoma (1), giant cell sarcoma (1), Kaposi sarcoma (1)

*p* < 0.05 are denoted in bold

**Table 2.** Preoperative management and oncologic outcomes after surgical treatment in reference centre.

	Primary resection *n* = 49	Referred *n* = 53	Univariate analysis *p*
Preoperative biopsy, *n* (%)			
°Yes	47 (95.9)	2 (3.7)	**< 0.00001**
°No	2 (4.1)	47 (88.6)	
°Unknown	0	4 (7.5)	
Discussion in MDTB, *n* (%) °Yes °No Margins of first surgery, *n* (%)	48 (97.9) 1 (2.1)	2 (3.7) 51 (96.2)	**< 0.00001**
°R0	46 (93.8)	3 (5.6)	**< 0.00001**
°R1	3 (6.1)	13 (24.5)	**0.01**
°R2/Rupture °Not informed	0 0	18 (33.9) 10 (18.8)	**< 0.00001** **0.001**
°Unknown	0	9 (16.9)	**0.002**
LR, *n* (%)			
°Yes	5 (10.2)	14 (26.4)	**0.04**
°No	44 (89.7)	39 (73.5)	
Distant metastasis, *n* (%)			
°Yes	13 (26.5)	10 (18.8)	0.4
°No	36 (73.5)	43 (81.1)	
First recurrence, *n* (%)			
°LR	4 (8.1)	13 (24.5)	**0.03**
°DM	13 (26.5)	8 (15.1)	0.2

MDTB = Multidisciplinary tumour board; LR = Local recurrence; DM = Distant metastasis

*p* < 0.05 are denoted in bold

**Table 3.** LR.

	No LR *n* = 58	LR *n* = 44	Univariate analysis *p*
Initial management, *n* (%) °Primary resection at tertiary center °Referred* °Age, median (IQR) Preoperative biopsy, *n* (%)	44 (75.8) 13 (22.4) 54 (44–70)	5 (11.3) 40 (90.9) 48 (39–67)	**< 0.00001** 0.4
°Yes	43 (74.1)	6 (13.6)	**< 0.00001**
°No	13 (22.4)	36 (81.8)	**< 0.00001**
°Unknown	1 (1.7)	3 (6.8)	0.3
Histopathology, *n* (%) °WDLPS °DDLPS °MLPS °LMS °UPS °MFS °Other sarcomas Margin status at first surgery, *n* (%)	7 (12.1) 9 (15.5) 7 (12.1) 14 (24.1) 4 (6.8) 7 (12.1) 10 (17.2)	1 (2.2) 4 (9.1) 4 (9.1) 6 (13.6) 11 (25) 4 (9.1) 14 (31.8)	0.1 0.3 0.7 0.2 **0.02** 0.7 0.1
°R0	40 (68.9)	6 (13.6)	**< 0.00001**
°R1	9 (15.5)	9 (20.4)	0.6
°R2/Rupture °Not informed/Unkown	3 (5.1) 4/1 (8.6)	15 (34.1) 6/8 (31.8)	**0.0002** **0.004**
Tumor size, median in cm (IQR)	6.8 (3.5–12.4)	4.75 (2.9–9.25)	0.1
°< 5 cm, *n* (%)	22 (37.9)	23 (52.2)	0.1
°5–10 cm, *n* (%) °>10 cm, *n* (%) °Not informed, *n* (%)	16 (27.5) 19 (32.7) 0	11 (25) 10 (22.7) 1 (2.2)	0.8 0.3 0.4
FNCLCC grade, *n* (%)			
°1	20 (34.4)	10 (22.7)	0.2
°2 °3 °Not informed	16 (27.5) 17 (29.3) 4 (6.8)	9 (20.4) 19 (43.1) 7 (15.9)	0.4 0.2 0.2
Adherence to sarcoma guidelines, *n* (%)			
°Yes °No	43 (74.1) 14 (24.1)	4 (9.1) 41 (93.1)	**< 0.00001**
Perioperative RT, *n* (%) °Preoperative RT °Postoperative RT	6 (10.3) 12 (20.6)	0 19 (43.1)	**0.03** **0.01**

* *For patients immediately referred due to whoops surgery (*n* = 20), final margin status of re-excision surgery considered (R0: 20/20)

WDLPS = Well differentiated liposarcoma; DDLPS = Dedifferentiated liposarcoma; MLPS = Myxoid liposarcoma; LMS = Leiomyosarcoma; UPS = Undifferentiated pleomorphic sarcoma; MFS = Myxofibrosarcoma; FNCLCC = French Federation of Cancer Centers Sarcoma Group; RT = Radiotherapy

Others = Malignant Peripheral Nerve Sheath Tumour (3), Fibrous solitary tumour (3), Fibromixoid sarcoma (3), synovial sarcoma (3), angiosarcoma (2), hemangiosarcoma (2), rhabdomyosarcoma (1), epithelioid sarcoma (1), myofibroblastic sarcoma (1), mixoinflamatory fibroblastic sarcoma (1), dermatofibrosarcoma (1), clear cell sarcoma (1), giant cell sarcoma (1), Kaposi sarcoma (1)

*p* < 0.05 are denoted in bold

**Table 4.** Multivariate analysis for LR.

			95% Confidence interval	
Predictor	*p*-value	Odds ratio	Lower	Upper
Positive margins	**< 0.001**	23.188	6.6693	80.618
Grade	0.11	2.493	0.7991	7.779
Size	0.93	1.004	0.9021	1.118
Perioperative RT	0.06	0.341	0.1096	1.059

*p* < 0.05 are denoted in bold

**Table 5.** Distant metastasis.

	No DM *n* = 77	DM *n* = 25	Univariate analysis *p*
Age, median (IQR) Initial management location, *n* (%) °At tertiary center °Referred Preoperative biopsy, *n* (%)	54 (42–68) 35 (45.4) 42 (54.5)	53 (45–68) 13 (52) 12 (48)	0.6 0.6
°Yes	35 (45.4)	13 (52)	0.6
°No	41 (53.2)	9 (36)	0.1
°Unknown	1 (1.2)	3 (12)	**0.04**
Histopathology, *n* (%) °WDLPS °DDLPS °MLPS °LMS °UPS °MFS °Other sarcomas Margins of first surgery, *n* (%)	8 (10.3) 7 (9.1) 10 (12.9) 12 (15.5) 10 (12.9) 11 (14.2) 19 (24.6)	0 6 (24) 1 (4) 8 (32) 5 (20) 0 5* (20)	0.1 0.08 0.2 0.08 0.5 0.06 0.7
°R0	52 (67.5)	16 (64)	0.7
°R1	9 (11.6)	2 (8)	0.7
°R2/Rupture °Not informed/Unknown	7 (9.1) 4/5 (11.6)	5 (20) 1/1 (8)	0.1 1
Tumor size, median in cm (IQR)	6 (3-12)	5 (3.5-12)	0.9
°< 5 cm, *n* (%)	35 (45.4)	9 (36)	0.4
°5–10 cm, *n* (%) °>10 cm, *n* (%) °Not informed, *n* (%)	19 (24.6) 22 (28.5) 1 (1.2)	8 (32) 8 (32) 0	0.6 0.8 1
FNCLCC grade, *n* (%)			
°1	29 (37.6)	1 (4)	**0.0008**
°2 °3 °No data	17 (22.1) 21 (27.2) 10 (12.9)	8 (32) 15 (60) 1 (4)	0.4 **0.004** 0.2
Adherence to sarcoma guidelines, *n* (%)			
°Yes	33 (42.8)	13 (52)	0.4
°No	44 (57.1)	12 (48)	

* Other sarcomas with DM: MPNST (2), angiosarcoma (1), G3 clear cell sarcoma (1), synovial sarcoma (1)

WDLPS = Well differentiated liposarcoma; DDLPS = Dedifferentiated liposarcoma; MLPS = Myxoid liposarcoma; LMS = Leiomyosarcoma; UPS = Undifferentiated pleomorphic sarcoma; MFS = Myxofibrosarcoma; FNCLCC = French Federation of Cancer Centers Sarcoma Group

Others = Malignant Peripheral Nerve Sheath Tumour (3), Fibrous solitary tumour (3), Fibromixoid sarcoma (3), synovial sarcoma (3), angiosarcoma (2), hemangiosarcoma (2), rhabdomyosarcoma (1), epithelioid sarcoma (1), myofibroblastic sarcoma (1), mixoinflamatory fibroblastic sarcoma (1), dermatofibrosarcoma (1), clear cell sarcoma (1), giant cell sarcoma (1), Kaposi sarcoma (1)

*p* < 0.05 are denoted in bold
